# Folic acid supplementation increases survival and modulates high risk HPV-induced phenotypes in oral squamous cell carcinoma cells and correlates with *p53 *mRNA transcriptional down-regulation

**DOI:** 10.1186/1475-2867-12-10

**Published:** 2012-03-23

**Authors:** Michael Moody, Oanh Le, Megan Rickert, Jeremy Manuele, Sarah Chang, Gary Robinson, Jeffrey Hajibandeh, John Silvaroli, Mark A Keiserman, Christine J Bergman, Karl Kingsley

**Affiliations:** 1 Department of Biomedical Sciences, School of Dental Medicine, University of Nevada, Las Vegas, USA; 2 School of Life Sciences, College of Sciences, University of Nevada, Las Vegas, USA; 3 College of Dental Medicine, Columbia University, New York, USA; 4 College of Sciences, University of Nevada, Reno, USA; 5 School of Health Related Professions: Nutrition, University of Medicine and Dentistry, New Jersey, USA; 6 Department of Food and Beverage, Harrah Hotel College, University of Nevada, Las Vegas, USA

**Keywords:** Folate, Human papillomavirus, Oral cancer

## Abstract

**Background:**

Although the primary risk factors for developing oral cancers are well understood, less is known about the relationship among the secondary factors that may modulate the progression of oral cancers, such as high-risk human papillomavirus (HPV) infection and folic acid (FA) supplementation. This study examined high-risk HPV and FA supplementation effects, both singly and in combination, to modulate the proliferative phenotypes of the oral cancer cell lines CAL27, SCC25 and SCC15.

**Results:**

Using a comprehensive series of integrated *in vitro *assays, distinct effects of HPV infection and FA supplementation were observed. Both high-risk HPV strains 16 and 18 induced robust growth-stimulating effects in CAL27 and normal HGF-1 cells, although strain-specific responses were observed in SCC25 and SCC15 cells. Differential effects were also observed with FA administration, which significantly altered the growth rate of the oral cancer cell lines CAL27, SCC15, and SCC25, but not HGF-1 cells. Unlike HPV, FA administration induced broad, general increases in cell viability among all cell lines that were associated with *p53 *mRNA transcriptional down-regulation. None of these cell lines were found to harbor the common C677T mutation in methylenetetrahydrofolate reductase (*MTHFR*), which can reduce FA availability and may increase oral cancer risk.

**Conclusion:**

Increased FA utilization and DNA hypermethylation are common features of oral cancers, and in these cell lines, specifically. The results of this study provide further evidence that FA antimetabolites, such as Fluorouracil (f5U or 5-FU) and Raltitrexed, may be alternative therapies for tumors resistant to other therapies. Moreover, since the incidence of oral HPV infection has been increasing, and can influence oral cancer growth, the relationship between FA bioavailability and concomitant HPV infection must be elucidated. This study is among the first pre-clinical studies to evaluate FA- and HPV-induced effects in oral cancers, both separately and in combination, which provides additional rationale for clinical screening of HPV infection prior to treatment.

## Background

Oral cancers take many years or decades to develop and may involve many separate, but inter-related processes [1,2]. Epidemiologic studies have provided evidence that tobacco and alcohol use are the major oral cancer risk factors, because of their direct and indirect carcinogenic effects on oral tissues [3,4]. During this lengthy process of carcinogenesis, many other factors are known to interact with, and modulate, oral tumor growth, including diet and infectious agents [5,6].

For example, a growing body of evidence has demonstrated that human papillomavirus (HPV) is a separate, independent oral cancer risk factor [7,8]. The high-risk HPV types involved in cervical carcinogenesis, HPV16 and HPV18, are present in a significant subset of oral cancers, and may contribute to oral carcinogenesis by similar mechanisms [7,9,10]. More specifically, the HPV "early" oncoproteins, E6 and E7, which promote viral replication, also bind *p53 *and disrupt tumor suppressor functions of retinoblastoma (*Rb*) and *Bcl-2 *[11]. Thus, infected cells bypass traditional G1/S cell cycle checkpoints disrupting the normal cell cycle. This disruption ultimately propels cell proliferation and drives carcinogenesis. More recent evidence also demonstrates HPV infection may function to modulate the growth of already existing oral tumors [12,13].

Despite understanding the primary mechanisms of HPV-mediated carcinogenesis [14], less is known about the secondary factors that modulate this transformation. A critical factor of HPV progression is methylation of the HPV genome [15,16]. Site-specific CpG methylation, mediated in part by adequate methyl donor availability (folate sufficiency), may be sufficient to slow or suppress HPV-driven carcinogenesis [17,18]. Partial demethylation or hypomethylation, which may result from inadequate methyl donor availability (folate insufficiency), is now known to be required for HPV-mediated cellular transformation [19].

Dietary folate intake facilitates specific metabolic processes, including the formation of S-adenosylmethionine, the primary methyl donor for DNA methylation reactions [20,21]. Human folate deficiencies are associated with many health disorders, including neural tube defects, vascular disease, microcytic anemia, and many types of cancer - including oral cancers [22-25]. Insufficient dietary intake of folate may result in dysregulation of DNA methylation, interfering with DNA synthesis and repair, which may initiate or trigger these adverse health conditions [26,27]. Some evidence now demonstrates that tobacco and alcohol use modulate folate metabolism by interfering with folate absorption and increasing renal excretion of folate, thereby lowering bioavailable folate [5,6,28]. In addition, previous studies also demonstrate that a common polymorphism in the methylenetetrahydrofolate reductase (*MTHFR*) gene, coding for the enzyme that produces the circulating form of folate, decreases function and capacity of this enzyme while increasing oral cancer risk [29,30].

To reduce the incidence of neural tube defect pregnancies associated with folate deficiency, the US Food and Drug Administration (FDA) adopted requirements for folate fortification of many food products starting in 1996 [31]. These measures were associated with an increase in mean dietary folate intake within the population, and a reduced incidence of neural tube defects and other folate deficiency-associated health conditions [32]. However, although folate sufficiency protects normal, non-neoplastic cells from turning cancerous through maintainance of normal DNA synthesis, repair and methylation, an opposite effect of supplementation on pre-existing early-stage colorectal cancers has been noted [33,34]. Most recently, some evidence now suggests that folate supplementation may also drive the growth and proliferation of oral cancers [35,36].

A thorough investigation of the inter-connected and inter-related mechanisms of the secondary factors that may modulate the growth and progression of oral cancers, such as high-risk HPV infection and folate supplementation is needed to elucidate these relationships. The purpose of this study is to elucidate the potential for high-risk HPV and folate supplementation, both singly and in combination, to modulate the proliferative phenotypes of well-characterized oral cancer cell lines.

## Materials and methods

### Cell culture

The human OSCC cell lines used in this study, CAL27 (CRL-2095), SCC15 (CRL-1623), SCC25 (CRL-1628), and HGF-1 (CRL-2014) were obtained from American Type Culture Collection (ATCC: Manassas, VA). CAL27 cells were maintained in Dulbecco's Modified Eagle's Medium (DMEM) with 4 mM L-glutamine, adjusted to contain 3.7 g/L sodium bicarbonate and 4.5 g/L glucose from Hyclone (Logan, UT). SCC15 and SCC25 cells were maintained in a 1:1 mixture of DMEM and Ham's F12 medium with 2.5 mM L-glutamine, modified to contain 15 mM HEPES, 0.5 mM sodium pyruvate, and 1.2 g/L sodium bicarbonate (ATCC), supplemented with 400 ng/ml hydrocortisone from Sigma-Aldrich (St. Louis, MO). The control oral cell line HGF-1 (CRL-2014) was maintained in DMEM with 4 mM L-glutamine, adjusted to contain 3.7 g/L sodium bicarbonate and 4.5 g/L glucose, from Hyclone (Logan, UT). Media for all cell lines was supplemented with 10% fetal bovine serum (FBS), and with 1% Penicillin (10,000 units/mL)-Streptomycin (10,000 μg/mL) solution (HyClone). Cell cultures were maintained in 75 cm^2 ^Becton, Dickinson (BD) Falcon tissue-culture treated flasks (Bedford, MA) at 37°C and 5% CO_2 _in humidified chambers.

### Materials

Folic acid (FA) with molecular weight (MW) = 441.40 was obtained from Fisher Bioreagents (Fair Lawn, New Jersey. Lot#075677). Proliferation and viability assays were performed in the appropriate complete media, with and without the addition of FA (100 μg/mL, 400 μg/mL). These concentrations represent 0.23 mM and 0.91 mM FA, supraphysiologic concentrations which allow for short-term *in vitro *effects to be observed.

100 μg/mL = 100 mg/L = 0.1 g FA/441.4 MW = 0.0002265 or 0.23 mM400 μg/mL = 400 mg/L = 0.4 g FA/441.4 MW = 0.0009062 or 0.91 mM

The US population mean folate serum concentration range is 2.7 nM/L and 11.4 nM/L, which corresponds with low-range and average US population folic acid equivalents intake measures, as of 2005 (79-376 μg/mL) [37]. The media used for these experiments, DMEM or Ham's F-12, with 10% FBS each contain 2.3 μM FA or the equivalent of 1.015 μg/mL, which is approximately 0.25 - 1% of the experimental concentrations used [38].

### Transfection

CAL27, SCC15, SCC25, and HGF-1 cells were seeded in 25 cm^2 ^BD Falcon tissue-culture treated flasks in appropriate media as described above and allowed to achieve 70% confluence. Cells were then transiently transfected by adding 1 μg/mL of the full-length HPV type 16, cloned into the pBluescript SK-vector (ATCC #45113) or the HPV type 18, cloned into the pBR322 vector (ATCC #45152). The transfections were performed using the Stratagene Mammalian Transfection Kit (La Jolla, CA) according to the manufacturer's recommended protocol for CaPO_4 _transfection. Mock transfectants (mTF) of each cell line were also established by performing the same transfection protocol, but without using virus. Transfections were confirmed by RT-PCR analysis of HPV mRNA production using extracted cellular RNA.

### Proliferation

Proliferation assays were performed using cell lines, transfected with HPV16, HPV18, and non-transfected controls, with and without the addition of FA (100 μg/mL, 400 μg/mL). Proliferation assays were performed in the appropriate complete media in Corning Costar 96-well assay plates (Corning, NY) at a concentration of 1.2 × 10^4 ^cells per well, and proliferation was measured over three days. Cultured cells were fixed at three time points, after 24 hrs (day 1), after 48 hrs (day 2), and after 72 hrs (day 3) using 50 μL of 10% buffered formalin, and were stained with crystal violet 1% aqueous solution (Fisher Scientific: Fair Lawn, NJ). The relative absorbance was measured at 630 nm using a Bio-Tek ELx808 microplate reader (Winooski, VT). Data were analyzed and graphed using Microsoft Excel (Redmond, WA) and SPSS (Chicago, IL). Three separate, independent replications of each experiment were performed.

### Relative-fold increase in proliferation

Trypsinizing and plating cells may have proliferation-stimulating effects within laboratory cell culture-based assays, which have been observed between d0 and d1 in previously published work involving this specific method of proliferation assay in these cell lines [36,39]. To reduce the overall impact of these effects, the relative change in proliferation, measured as the change or relative-fold increase (RFI) in absorbance between d3 and d1, can be calculated to more accurately assess the changes induced by these experimental treatments.

### Statistics

Comparisons of the effects of treatments were made using two-tailed *t *tests with α = .05. All samples were analyzed using two-tailed *t *tests as departure from normality can make more of a difference in a one-tailed than in a two-tailed *t *test [40]. As long as the sample size is even moderate (> 20) for each group, quite severe departures from normality make little practical difference in the conclusions reached from these analyses. To confirm the effects observed from these experiments, further analysis of the data was facilitated with ANOVA using SPSS (Chicago, IL). Significance for ANOVA was *p *< 0.05.

### Survival and viability

Prior to plating cells for proliferation assays, aliquots of trypsinized cells were stained using Trypan Blue (Sigma: St. Louis, MO), and live cells were enumerated by counting the number of Trypan-blue negative cells using a VWR Scientific Counting Chamber (Plainfield, NJ) and a Zeiss Axiovert 40 inverted microscope (Gottingen, Germany). At each time point (d1-d3), several wells were processed using the Trypan stain, and live cells were enumerated using this procedure.

### DNA isolation, PCR, and RFLP

To determine if these cell lines harbored the most common DNA polymorphism in the *MTHFR *gene (C677T) or any endogenous HPV virus, DNA was isolated from 1.5 × 10^7 ^cells from each cell line using the GenomicPrep DNA isolation kit (Amersham Biosciences: Buckinghamshire, UK), using the procedure recommended by the manufacturer. DNA concentration was measured using the optical density absorbance value measured by a spectrophotometer at 260 nm. DNA purity was calculated using ratio measurements of absorbance at 260 and 280 nm; A260:A280 purity range should be between 1.7 and 2.0. DNA from each cell line was used to perform polymerase chain reaction (PCR) with the Fisher exACTGene complete PCR kit (Fisher Scientific: Fair Lawn, NJ) and a Mastercycler gradient thermocycler (Eppendorf: Hamburg, Germany) using the following *MTHFR *[41], and HPV [13,35] primers, synthesized by SeqWright (Houston, TX):

*MTHFR *677 forward primer, 5'-TGAAGGAGAAGGTGTCTGCGGGA-3'

*MTHFR *677 reverse primer, 5'-AGGACGGTGCGGTGAGAGTG'3'

HPV18 forward primer, ATGGCGCGCTTTGAGGATCC;

HPV18 reverse primer, GCATGCGGTATACTGTCTCT;

HPV16 forward primer, ATGTTTCAGGACCCACAGGA;

HPV16 reverse primer, CCTCACGTCGCAGTAACTGT;

One μg of template DNA was used for each reaction. The initial denaturation step ran for 1 minute at 94°C. Thirty amplification cycles were run, consisting of 30 second denaturation at 94°C, 60 seconds of annealing at 58°C, and 6.5 minutes of extension at 68°C. Final extension was run for 5 minutes at 68°C. No cell lines were found to harbor HPV16 or HPV18 DNA.

The C677T *MTHFR *polymorphism creates a *Hinf1 *cleavage site, yielding DNA fragment lengths of 175 bp and 23 bp in the presence of the variant T allele. Thus the restriction fragment length polymorphisms (RFLP) or 677 *MTHFR *genotype banding patterns are as follows; wild type (C/C) 198 bp, heterozygote (C/T) 198 bp, 175 bp and 23 bp, and homozygote recessive 175 bp and 23 bp [42,43]. The *MTHFR *PCR reaction products (fragment length 198 bp) were therefore digested overnight at 37°C using the restriction enzyme *HinfI *(New England Biolabs: Ipswich, MA) and the reaction products were separated by gel electrophoresis using Reliant 4% NuSieve^® ^3:1 Plus Agarose gels (Lonza: Rockland, ME). Bands were visualized by UV illumination of ethidium-bromide-stained gels and captured using a Kodak Gel Logic 100 Imaging System and 1D Image Analysis Software (Eastman Kodak: Rochester, NY).

### RT-PCR

To determine the cellular phenotype of *p53 *mRNA transcription, as well as to confirm HPV-specific mRNA production post-transfection, RNA was isolated from 1.5 × 10^7 ^cells after HPV transfection and also following FA administration using ABgene Total RNA Isolation Reagent (Epsom: Surrey, UK) and the procedure recommended by the manufacturer. RT-PCR was performed with the ABgene Reverse-iT One-Step RT-PCR Kit ReadyMix Version and a Mastercycler gradient thermocycler (Eppendorf: Hamburg, Germany) using primers for the control, Glyceraldehyde 3-phosphate dehydrogenase (*GAPDH*) [44], and *p53 *[45], as well as for HPV16 and 18:

*p53 *forward primer, 5'-ACCAGGGCAGCTACGGTTTC'3'

*p53 *reverse primer, 5'-CCTGGGCATCCTTGAGTTCC-3'

*GAPDH *forward primer, ATCTTCCAGGAGCGAGATCC;

*GAPDH *reverse primer, ACCACTGACACGTTGGCAGT;

One μg of template (total) RNA was used for each reaction. The reverse transcription step ran for 30 minutes at 47°C, followed by denaturation for 2 minutes at 94°C. Thirty-five amplification cycles were run, consisting of 20 second denaturation at 94°C, 30 seconds of annealing at 58°C, and 6.5 minutes of extension at 72°C. Final extension was run for 5 minutes at 72°C. Reaction products were separated by gel electrophoresis using Reliant 4% NuSieve^® ^3:1 Plus Agarose gels (Lonza: Rockland, ME). Bands were visualized by UV illumination of ethidium-bromide-stained gels and captured using a Kodak Gel Logic 100 Imaging System and 1D Image Analysis Software (Eastman Kodak: Rochester, NY). Quantitation of RT-PCR band densitometry was performed using Adobe (San Jose, CA) Photoshop imaging software, Image Analysis tools.

## Results

**Figure 1:**
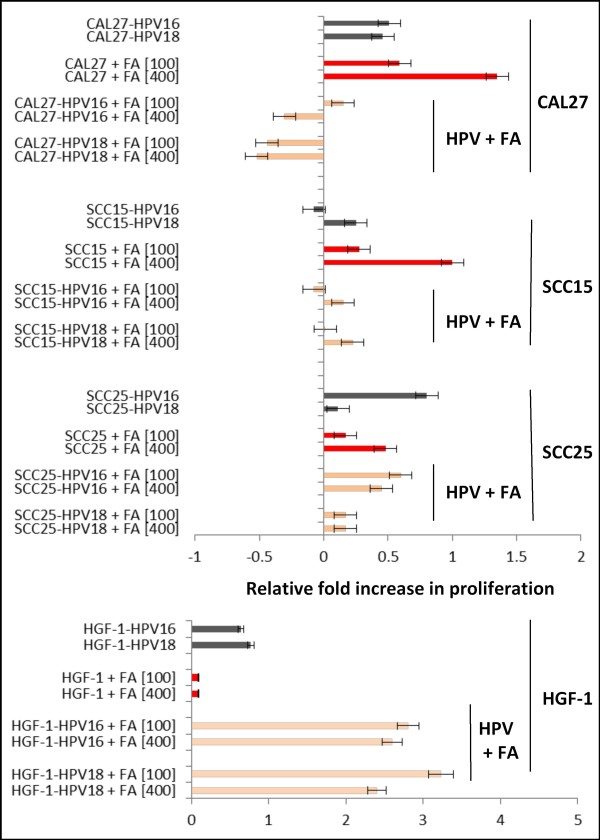
**Measurement of relative-fold increase (RFI): FA modulation of HPV effects**. Analysis of RFI in CAL27 cells revealed the growth stimulating effects of HPV16, HPV18, FA [100] and FA [400] are reduced or inhibited when HPV and FA are concomitantly administered. Similarly, SCC15 cell growth stimulated by HPV18, FA [100 μg/mL] and FA [400 μg/mL] is either reduced or inhibited by concomitant HPV and FA administration. SCC25 cell growth, stimulated by HPV16, FA [100 μg/mL] and FA [400 μg/mL] is also significantly reduced, although not completely inhibited by concomitant HPV and FA administration. HGF-1 cell growth, stimulated by HPV16 and HPV18 but not FA administration, was robustly enhanced by concomitant administration of HPV and FA. Both HPV16 and HPV18 enhanced HGF-1 proliferation at either concentration of FA [100 or 400 μg/mL].

The oral squamous cell carcinoma lines, CAL27, SCC25, and SCC15, were grown in 96-well assay plates over three days in separate, independent experiments to establish baseline measurements of growth among untreated cells (Figure 1). All three cell lines rapidly increased in confluence more than four-fold over three days. The growth of the normal, non-tumorigenic cell line, HGF-1, increased slightly more than two-fold, from less than 10% confluence to approximately 22% during the same period. To reduce the proliferation-stimulating effects of trypsinizing and plating cells, previously observed as an initial cell division observed between d0 and d1 in these cell lines [13,36,46,47], the RFI, measured as the absorbance value at d3 minus the value at d1, was evaluated. This analysis revealed that the proliferation of CAL27 cells was mainly observed between d1 and d3, with HPV16, HPV18, FA [100 μg/mL] and FA [400 μg/mL]. However, the combined effects of FA and HPV were then assessed, which revealed that the concomitant addition of FA at either concentration significantly inhibited HPV-mediated growth (*p *< 0.05).

More specifically, CAL27 HPV16-mediated growth was reduced by FA administration at the lower concentration (100 μg/mL) to 0.15-fold over baseline growth and inhibited to -0.31-fold at the higher concentration and GS_MAX _(400 μg/mL). Similarly, HPV18-mediated growth of CAL27 cells was inhibited by FA administration at these concentrations to -0.45 and -0.55-fold, respectively (*p *< 0.05).

Similarly, SCC15 proliferation was mainly observed between d1 and d3, with HPV16, HPV18, FA [100 μg/mL] and FA [400 μg/mL]. However, the combined effects of HPV16 and FA at 100 μg/mL did not stimulate SCC15 growth (-0.075-fold, *p *> 0.05), but did at 400 μg/mL (+0.15-fold, *p *< 0.05). The combination of HPV18 and FA at 100 μg/mL also did not stimulate SCC15 growth (0.01-fold, *p *> 0.05), but did at 400 μg/mL (+0.225-fold, *p *< 0.05).

Finally, the combined effects of HPV16 and FA on SCC25 cells at 100 μg/mL strongly enhanced growth (0.6-fold, *p *< 0.05), as did FA at 400 μg/mL (0.45-fold, *p *< 0.05). The combined effects of HPV18 and FA on SCC25 cells at either 100 or 400 μg/mL were similar, increasing growth modestly (0.15-fold, *p *< 0.05)

Intriguingly, the effects of HPV16 and HPV18 on HGF-1 cell proliferation were strongly enhanced by FA administration. FA supplementation increased HPV16-mediated HGF-1 growth by 2.8-fold and 2.6-fold at the lower and higher concentrations evaluated (*p *< 0.05), while HPV18-mediated growth was increased by 3.2-fold and 2.4-fold at these concentrations (*p *< 0.05).

### Survival and viability

To determine if the observed effects on cell growth induced by HPV transfection or FA supplementation were due, in part, to changes in cell survival, each cell line and experimental treatment were analyzed at each time point of the proliferation assays and under each of the treatment conditions (Table 1). The addition of HPV16 or HPV18 increased survival among CAL27 cells from an average of 73% in non-transfected cells to 94% and 95%, respectively. Significant increases in survival were also observed under both high and low concentrations of FA.

**Table 1:** Cell viability

	CAL27	SCC15	SCC25	HGF-1
	73%	79%	80%	84%

HPV16	**94%**	80%	**86%**	**94%**

HPV18	**95%**	**88%**	79%	**93%**

FA [100 μg/mL]	**96%**	**94%**	**97%**	**91%**

FA [400 μg/mL]	**96%**	**91%**	**99%**	**93%**

SCC15 baseline survival (79%) was relatively unaffected by HPV16 (80%), but was significantly enhanced HPV18 (88%). However, significant increases in survival were observed under both conditions of FA administration, to 94% (100 μg/mL) and 91% (400 μg/mL). Although HPV16 increased SCC25 survival to 86% from a baseline of 80%, no such increase in survival observed with HPV18 (79%). FA administration was associated with significant increases in SCC25 survival. Survival among HGF-1 cells (84%) was also significantly increased by HPV16, HPV18 and FA treatments by nearly equal levels (~10%).

### MTHFR RFLP screening

**Figure 2:**
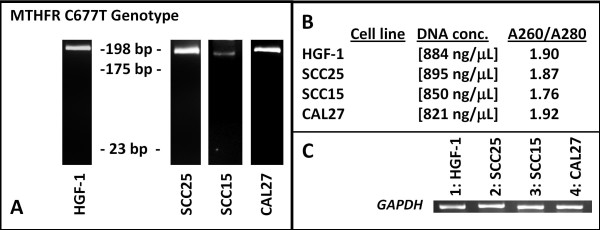
**DNA and RFLP analysis**. A) *Hinf*1 restriction digestion (RFLP) of PCR products (C/C: 198 bp) were used to screen for C677T SNP genotype of MTHFR. The C677T MTHFR polymorphism creates a *Hinf*1 cleavage site, yielding DNA fragment lengths of 175 bp and 23 bp in the presence of the variant T allele. The normal (HGF-1) and all three oral cancer cell lines (SCC25, SCC15, CAL27) were found to harbor wild type (C/C) genetic profiles; no heterozygotes (C/T) or homozygous mutants (T/T) were observed. B) DNA concentration and purity derived from absorbance readings at 260 and 280 nm revealed similar concentrations (821-895 ng/μL) and purity (1.76 - 1.92) from each cell line. C) PCR using control using GAPDH established similar band intensities from each cell line.

Because folate bioavailability may be reduced by a common DNA single nucleotide polymorphism (SNP) in the methylenetetrahydrofolate reductase (*MTHFR*) gene, which encodes the enzyme responsible for producing the circulating form of folate, each cell line was examined to determine if the most common *MTHFR *DNA polymorphism (C677T) was present. The RFLP analysis demonstrated that CAL27, SCC25, SCC15 and HGF-1 cells were not found to contain the *MTHFR *C677T polymorphism (Figure 2A). To rule out confounding effects of DNA concentration, absorbance measurements were taken, revealing similar DNA concentrations from each cell line, which ranged from 821 - 895 ng/μL (Figure 2B). Analysis of A260/A280 ratio confirmed the similarity of purity from the DNA isolates, which averaged between 1.76 and 1.92. PCR amplifying the housekeeping gene, *GAPDH*, revealed nearly identical band patterns of similar intensity (Figure 2C).

### p53 mRNA transcription

**Figure 3:**
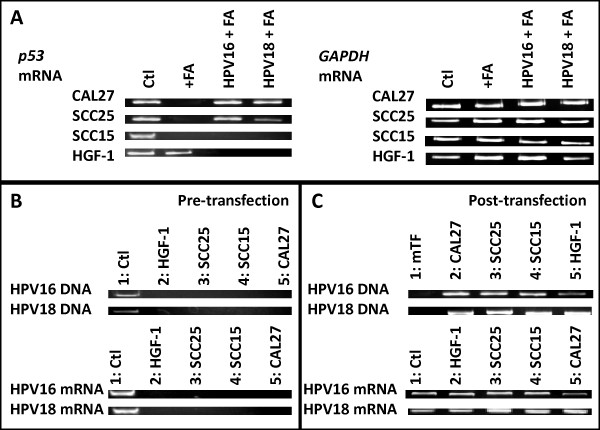
**DNA and mRNA analysis**. A) RT-PCR performed on total RNA extracted from cells at d1 (24 hr) following FA administration [400 μg/mL] revealed little change in p53 mRNA transcription in HGF-1 cells, but decreased transcription in CAL27, SCC15, and SCC25 cells. HPV16 and HPV18 had little effect on FA-induced changes in p53 transcription in CAL27 and SCC25 cells, although decreases were observed in both SCC15 and HGF-1 cells. B) PCR screening from DNA confirmed HPV16 and HPV18 DNA was present in the control cell lines, CaSKi (HPV16) and GH354 (HPV18) cervical adenocarcinomas, but not HGF-1, SCC25, SCC15 or CAL27. RT-PCR screening also confirmed the presence of HPV-specific mRNA in the control, but not experimental, cell lines. C) DNA screening post-transfection confirmed the presence of HPV-specific DNA in CAL27, SCC25, SCC15 and HGF-1, but not mock transfectants (mTF); RNA screening confirmed production of HPV-specific mRNA at levels similar to control cell lines.

Some evidence has demonstrated the differing effects of folic acid supplementation according to the *p53 *transcription profile of the tumor. The *p53 *mRNA transcription profile for each cell line under each experimental condition, untreated (control: Ctl), FA at 400 μg/mL (+FA), HPV16 or HPV18 + FA was examined (Figure 3A). Densitometric measurements of relative end-point RT-PCR band intensity revealed administration of FA significantly reduced *p53 *mRNA transcription in CAL27, SCC25, and SCC15 cells nearly completely. HGF-1 mRNA transcription, however, was relatively unaffected (-5%).

The concomitant administration of HPV and FA also exhibited differential effects on *p53 *mRNA transcription in each cell line. In CAL27 cells, *p53 *mRNA transcription was restored and indistinguishable from untreated CAL27 cells. In SCC25 cells, however, HPV18 + FA was associated with more than three-fourths reduction in *p53 *mRNA transcription (-76%), while HPV16 + FA administration was associated with a more modest inhibition (-25%). Interestingly, *p53 *mRNA transcription in SCC15 cells was inhibited under all conditions evaluated, +FA, HPV16 + FA, and HPV18 + FA. The normal cell line, HGF-1, however exhibited no significant changes in *p53 *mRNA transcription under FA administration alone, although a nearly complete reduction under HPV16 + FA and HPV18 + FA was observed. *GAPDH *mRNA transcription in each cell line, however, remained relatively constant.

### HPV screening

Finally, to confirm these cell lines did not already harbor endogenous HPV, which could potentially confound these observations, DNA and RNA from each cell line was used to perform PCR and RT-PCR screenings using primers specific for HPV16 and HPV18 (Figure 3B and 3C). These results demonstrated that HGF-1, SCC25, SCC15 and CAL27 cells did not harbor HPV16 or HPV18 DNA (Figure 3B). DNA isolated from HPV-positive cervical cancer cell lines (CaSKi, HPV16; GH354, HPV18) was used as PCR positive controls. In addition, DNA presence post-transfection was confirmed using PCR, which demonstrated HPV-specific DNA was present each cell line, but not in the mock transfectants (mTF) (Figure 3C). In addition, HPV-specific mRNA was expressed in all cell lines following transfection, which was similar to mRNA expression levels in control cells.

## Discussion

Although epidemiologists and oral health researchers concur that the majority of oral cancers originate from the use of tobacco and alcohol, recent studies have also suggested that both HPV infection and folate sufficiency accelerated oral cancer growth [7,8,48,49]. The goal of this study was to investigate the possibility that high-risk HPV infection and FA administration, both singly and in combination, could mediate the proliferation of well-characterized oral cancers. To test these hypotheses, a comprehensive series of integrated *in vitro *assays were performed that clearly demonstrated distinct effects of HPV infection and FA supplementation in each cell line examined. For example, although both high-risk HPV strains induced robust growth-stimulating effects in CAL27 and HGF-1 cells, strain-specific responses were observed in both SCC25 and SCC15 cells. These results are consistent with previous reports of HPV strain-specific effects [13], as well as evidence that oral cancers are not dependent upon the inhibition of a single signal transduction or tumor suppression pathway for growth and development [50].

It is important to note that increased survival and viability was also observed under conditions of HPV-stimulated growth. These observations suggest that modulation of either *p53 *or *Rb*-specific pathways by HPV E6 and E7 genes could be responsible for lowering the barriers to G1/S cell cycle progression, thereby simultaneously increasing both the rates of proliferation as well as cell viability as previously observed [51-53]. Furthermore, that FA administration increased proliferation and simultaneously down-regulated *p53 *mRNA transcription in all three oral cancer cell lines, but not HGF-1 cells, strongly suggests this pathway may be, in part, critical to our understanding of these processes.

Another key finding was the observation of differential effects induced by FA administration, which significantly altered the growth rate of oral cancers, but not normal cells. This evidence suggests folate bioavailability may be a rate-limiting step among oral cancers that have impaired or reduced function in one or more cell cycle inhibition pathways, which is further supported by the observation that these dose-dependent effects can be saturated [36,54]. Unlike HPV, FA administration induced broad, general increases in cell viability among all cell lines, which may support the observations that folate bioavailability may also play critical roles in biosynthetic and epigenetic pathways that are not directly related to cellular growth or proliferation [22,29,30]. Although FA administration increased cellular viability in the normal cell line, this increase was comparatively smaller than the effects in the oral cancer cell lines, which might suggest these larger changes in survival and viability might function to exacerbate the observed proliferation-stimulating effects among oral cancers over time.

Previous studies have demonstrated the proliferation stimulating effects of HPV and FA administration on oral cancers may suggest that concomitant removal or reduction of *p53 *and *Rb *tumor suppression pathways and increased folate bioavailability could greatly amplify cellular growth [7,8,22,24,27]. In fact, this was observed in the responses of the normal cell line, HGF-1. However, the combined effects of HPV and FA administration were strikingly different, with a reduction or complete inhibition of growth observed in all three oral cancer cell lines. Further analysis revealed several studies that now confirm CpG site-specific methylation of HPV DNA, mediated in part by folate availability, is sufficient to suppress neoplastic progression [17,18,55,56]. Although preferential DNA methylation in *p53 *exons 248 and 273 by DNA methyltransferases may occur during oral carcinogenesis [57,58], CpG methylation of the HPV genome is among the most important factors that limit viral production and HPV-mediated phenotypes [15,16,59]. Based upon this understanding, a more thorough and complete investigation is warranted to explore the role of folate in mediating the availability of methyl groups for CpG-specific DNA methylation, influencing *p53 *and HPV mRNA transcription, as well as biosynthetic pathways in oral cancers.

Finally, a limitation of this and other preclinical studies involves the use of oral cancer cell lines, as there may be underlying dissimilar genetic mutations that might potentially influence the experimental outcomes. For example, the SCC25 cell line has been found to contain a deletion in the cdk1 promoter that contains a key transcriptional repressor region [60]. The CAL27 cell line contains a nonsense mutation in the SMAD4 gene, while SCC15 cells were found to harbor a missense mutation in SMAD2 - both signal TGF-β transduction proteins [61]. In addition, both CAL27 and SCC15 cells contains a single nucleotide polymorphism in the S100A2 gene, a calcium-binding tumor suppressor protein, although this did not appear to alter their propensity for growth, migration or invasion [62]. However, a growing body of evidence demonstrates that dysregulation and reduced expression of key tumor suppressors, such as p16, in these cell lines may, in fact, be the result of hypermethylation - providing further justification to elucidate the interconnected roles of FA bioavailability and utilization may play in the growth and progression of oral cancers [63-65].

## Conclusions

Although individual studies have suggested that either high-risk HPV infection or FA administration may influence the growth and progression of oral cancers, evidence is now emerging that indicates folate bioavailability and oral HPV infection are very closely inter-connected. The policy of folate food fortification, routine multivitamin use and FA supplementation, combined with an ever-increasing prevalence of oral HPV infections, suggests a more complete and thorough investigation of these inter-relationships is needed. This study is among the first to examine these relationships and provide specific information about *p53*-specific pathway modulation and *MTHFR *genotypes in oral cancers, which may be particularly useful to oncologists and oral health researchers as they develop rubrics for generalizing the effects and most effective treatment options for patients with oral cancer.

## Abbreviations

HPV: Human papillomavirus; *MTHFR*: methylenetetrahydrofolate reductase; *Rb*: retinoblastoma; FDA: US Food and Drug Administration; ATCC: American Type Culture Collection; DMEM: Dulbecco's Modified Eagle's Medium; FA: Folic acid; mTF: Mock transfectants; RFI: relative-fold increase; PCR: polymerase chain reaction; *GAPDH*: Glyceraldehyde 3-phosphate dehydrogenase; RFLP: restriction fragment length polymorphisms

## Competing interests

The authors declare that they have no competing interests.

## Authors' contributions

KK, MAK and CJB conceived and designed this project. MM, OK, MR, JM, SC, JH, JS, GR and KK were responsible for performing the experimental assays, data collection, figure generation, and writing. MAK and KK were responsible for editing the final manuscript. All authors read and approved the final manuscript.
